# Investigating the role of rare coding variability in Mendelian dementia genes (*APP*, *PSEN1*, *PSEN2*, *GRN*, *MAPT*, and *PRNP*) in late-onset Alzheimer's disease

**DOI:** 10.1016/j.neurobiolaging.2014.06.002

**Published:** 2014-12

**Authors:** Celeste Sassi, Rita Guerreiro, Raphael Gibbs, Jinhui Ding, Michelle K. Lupton, Claire Troakes, Safa Al-Sarraj, Michael Niblock, Jean-Marc Gallo, Jihad Adnan, Richard Killick, Kristelle S. Brown, Christopher Medway, Jenny Lord, James Turton, Jose Bras, Alzheimer's Research UK Consortium, Kevin Morgan, John F. Powell, Andrew Singleton, John Hardy

**Affiliations:** a Department of Molecular Neuroscience, UCL Institute of Neurology, University College London, London, UK; b Laboratory of Neurogenetics, National Institute on Aging, National Institutes of Health, Bethesda, MD, USA; c King's College London Institute of Psychiatry, London, UK; d Translation Cell Sciences-Human Genetics, School of Life Sciences, Queens Medical Centre, University of Nottingham, Nottingham, UK

**Keywords:** Alzheimer's disease, Neurodegenerative dementia, *APP*, *PSEN1*, *PSEN2*, *MAPT*, *GRN*, *PRNP*, Exome sequencing

## Abstract

The overlapping clinical and neuropathologic features between late-onset apparently sporadic Alzheimer's disease (LOAD), familial Alzheimer's disease (FAD), and other neurodegenerative dementias (frontotemporal dementia, corticobasal degeneration, progressive supranuclear palsy, and Creutzfeldt-Jakob disease) raise the question of whether shared genetic risk factors may explain the similar phenotype among these disparate disorders. To investigate this intriguing hypothesis, we analyzed rare coding variability in 6 Mendelian dementia genes (*APP*, *PSEN1*, *PSEN2*, *GRN*, *MAPT*, and *PRNP*), in 141 LOAD patients and 179 elderly controls, neuropathologically proven, from the UK. In our cohort, 14 LOAD cases (10%) and 11 controls (6%) carry at least 1 rare variant in the genes studied. We report a novel variant in *PSEN1* (p.I168T) and a rare variant in *PSEN2* (p.A237V), absent in controls and both likely pathogenic. Our findings support previous studies, suggesting that (1) rare coding variability in *PSEN1* and *PSEN2* may influence the susceptibility for LOAD and (2) *GRN*, *MAPT*, and *PRNP* are not major contributors to LOAD. Thus, genetic screening is pivotal for the clinical differential diagnosis of these neurodegenerative dementias.

## Highlights

• We have used exome sequencing to investigate rare coding variability in Mendelian dementia genes (*APP*, *PSEN1*, *PSEN2*, *GRN*, *MAPT*, and *PRNP*) in a cohort composed of 141 late-onset sporadic Alzheimer's disease cases and 179 elderly controls, autopsy proven from the UK.
• We report a novel mutation in *PSEN1* (p.I168T) and a rare variant in *PSEN2* (p.A237V), both likely pathogenic.
• We conclude that *PSEN1* and *PSEN2* harbor susceptibility factors for sporadic Alzheimer's disease. By contrast, *GRN*, *MAPT*, and *PRNP* do not play a major role for the development of late-onset sporadic Alzheimer's disease.
• Genetic screening is therefore pivotal for a clinical differential diagnosis of sporadic late-onset Alzheimer's disease and other neurodegenerative dementias (frontotemporal dementia, corticobasal degeneration, progressive supranuclear palsy, and Creutzfeldt-Jakob disease).

## 1 Introduction

Alzheimer's disease (AD) (OMIM #104310) is the most common cause of progressive dementia in the elderly individuals. Aging and genetic factors play a pivotal role for the disease development. AD incidence increases exponentially from the age of 65 years (1.5% affected) to 80 years and older (30% affected). Twin studies have shown that AD heritability ranges between 60% and 80% (Bergem et al., 1997, Gatz et al., 2006, Raiha et al., 1996). Fully penetrant mutations in amyloid precursor protein (*APP*) and presenilins (*PSEN1* and *PSEN2*) are known to cause familial autosomal dominant AD. The *APOE* ε4 allele is the main risk factor for apparently sporadic AD. In the last 5 years, genome-wide association studies (GWASs) identified several loci, harboring common variants with low risk effect size (OR: 1.2–1.5) (Harold et al., 2009, Hollingworth et al., 2011, Lambert et al., 2009, Lambert et al., 2013, Naj et al., 2011, Seshadri et al., 2010).

Recently, next generation sequencing has led to enormous progress in AD genetics, with the discovery of 2 rare significant risk factors, mapping to *TREM2* (p.R47H) and *PLD3* (p.V232M), and a very rare protective variant in *APP* (p.A637T) (Cruchaga et al., 2013, Guerreiro et al., 2013, Jonsson et al., 2012). In addition, *C9orf72* repeat expansion has been reported in a few patients with clinical AD (Majounie et al., 2012).

The overlapping clinical and neuropathologic features between AD and other neurodegenerative dementias (frontotemporal dementia [FTD], corticobasal degeneration [CBD], progressive supranuclear palsy [PSP], and Creutzfeldt-Jakob disease [CJD]) lead to a misdiagnosis in 17%–30% of AD cases (Beach et al., 2012). This raises the question of whether genetic risk factors relevant in such dementias may play a role in late-onset Alzheimer's disease (LOAD). GWASs have shown that common noncoding variability in Mendelian dementia genes (*APP*, *PSEN1*, *PSEN2*, *MAPT*, *GRN*, and *PRNP*) does not influence susceptibility to AD. By contrast, a growing body of evidence highlighted the significant role of rare coding variants in *PSEN1* in LOAD (Benitez et al., 2013, Cruchaga et al., 2012). Thus, to test the hypothesis that rare coding variability in genes relevant for familial Alzheimer's disease (FAD) and other types of dementia (*APP*, *PSEN1*, *PSEN2*, *MAPT*, *GRN*, and *PRNP*) may underlie LOAD pathogenesis, we have analyzed exome sequencing data, in a British cohort composed of 141 LOAD cases without any apparent family history and 179 elderly controls autopsy proven.

## 2 Methods

### 2.1 Cases and controls

Our cohort was composed of 141 independent LOAD (age at onset ≥65 years) cases and 179 elderly (>60 years) unrelated controls, neuropathologically confirmed. These patients were referred as apparently sporadic LOAD cases.

All the patients and controls were Caucasian, mostly from the UK (London, Manchester, Nottingham, and Edinburgh) and to a lesser extent from North America. The average age at diagnosis was 76.7 years (range 65–97 years) for the LOAD patients and the mean age of ascertainment was 78 years (range 60–102 years) for the controls (Table 1).

**Table 1:** Cohort

Cohort	n	Diagnosis	Sequencing strategy	Age (y) mean ± SD (range)	Male (%)	*APOE* e4+ (%)
LOAD CASES	141	Clinical and neuropathologic	Exome sequencing	76.7 (65–97)	42	62
CONTROLS	179	Clinical and neuropathologic	Exome sequencing	78 (60–102)	55	40.7

Key: LOAD, late-onset Alzheimer's disease; SD, standard deviation.

Written informed consent was obtained for each individual and the study was approved by the appropriate institutional review boards.

### 2.2 Exome sequencing

Library preparation for next-generation sequencing was performed according to the NimbleGen (Roche NimbleGen v2) and TruSeq (Illumina) sample-preparation protocols. DNA libraries were then hybridized to exome-capture probes with NimbleGen SeqCap EZ Human Exome Library, version 2.0 (Roche NimbleGen) or TruSeq (Illumina). Each capture method covers the *APP*, *PSEN1*, *PSEN2*, *GRN*, *MAPT*, and *PRNP* loci. Exome-enriched libraries were sequenced on the Illumina HiSeq 2000 using 2 × 100 bp paired end read cycles.

### 2.3 Bioinformatics

Sequence alignment and variant calling were performed against the reference human genome (UCSC hg19). Paired end sequence reads (2 × 100 bp paired end read cycles) were aligned using the Burrows-Wheeler aligner (Li and Durbin, 2009). Format conversion and indexing were performed with Picard (www.picard.sourceforge.net/index.shtml). The Genome Analysis Toolkit was used to recalibrate base quality scores, perform local realignments around indels and to call and filter the variants (McKenna et al., 2010). VCFtools was used to annotate gene information for the remaining novel variants. We used ANNOVAR software to annotate the variants (Wang et al., 2010). Variants were checked against established databases (1000 Genomes Project and dbSNP v.134). The protein coding effects of variants were predicted using SIFT, Polyphen2, and SeattleSeq Annotation (gvs.gs.washington.edu/SeattleSeqAnnotation). All variants within the coding regions of *APP*, *PSEN1*, *PSEN2*, *MAPT*, *GRN*, and *PRNP* were annotated for both cases and controls.

### 2.4 Sanger sequencing

All rare variants identified by whole exome sequencing in the candidate genes were validated by Sanger sequencing.

Primers for exons harboring rare variants were designed in Primer3 (http://bioinfo.ut.ee/primer3-0.4.0/) using UCSC (http://genome.ucsc.edu/) reference sequences NM_000484.3 (*APP*), NM_000021.3 (*PSEN1*), NM_000447.2 (*PSEN2*), NM_001123066.3 (*MAPT*), NM_002087.2 (*GRN*), and NM_000311.3 (*PRNP*).

Purified sequences were analyzed on an ABI 3730 DNA Analyzer (Applied Biosystems, CA, USA) and electropherograms were visualized in Sequencher software (version 4.2 Gene Codes Corporation, MI, USA).

### 2.5 Apoe genotyping

*APOE* genotypes comprising the *APOE* ɛ2, ɛ3, and ɛ4 alleles were assayed using the TaqMan method (Applied Biosystems Inc [ABI], Foster City, CA, USA). SNP-specific primers and probes were designed by ABI (TaqMan genotyping assays).

## 3 Results

We identified 226 variants (nonsynonymous, synonymous, intronic, and UTRs) and 18 indels (coding and intronic) in the genes studied. Of these, we analyzed the 18 rare coding variants (minor allele frequency <1%), 1 splice-site mutation (*MAPT* c.115–2A>T), 1 low frequency and 1 common coding polymorphisms in *PRNP*: a 24 bp deletion (rs138688873) and the p.M129V (rs1799990), respectively. In our cohort, 14 LOAD cases (10%) and 11 controls (6%) carry at least one of these rare variants (Table 2). We detected 5 novel variants: 3 present in cases (*APP* p.Y538H, *PSEN1* p.I168T, and *MAPT* c.115-2A>T) and 2 in controls (*MAPT* p.G200E and *PRNP* p.M134V).

**Table 2:** Rare variants found in *APP*, *PSEN1*, *PSEN2*, *MAPT*, *GRN*, *PRNP* in 141 LOAD cases and 179 controls

Variant interpretation	Gene	Position	Nucleotide change	Aa change	Minor allele	status	SIFT/Polyphen	LOAD cases (n = 141)				Comment	CONTROLS (n = 179)		
								Count (%)	(AAO–AAD)	Genotype	*APOE*		Count (%)	Genotype	*APOE*
PROBABLE PATHOGENIC*															
	*PSEN1*	14:73653583	c.503T>C	p.I168T	C	novel	possibly-damaging	1 (0.7)	86y-94y	T/C	ε2ε4	p.I168del reported in FAD	0	-	-
	*PSEN2*	1:227076673	c.710 C>T	p.A237V	T	rs200670135	possibly-damaging	1 (0.7)	87y-95y	C/T	ε3ε3	Homologous residue in *PSEN1* (p.A231)	0	-	-
*LIKELY RARE BENIGN POLYMORPHISMS*															
	*APP*	21:27423376	c.602 C>T	p.A201V	T	rs149995579	tolerated	0	-	-	-	EXON 5	1 (0.5)	C/T	ε3ε4
		21:27326979	c.1612 T>C	p.Y538H	C	novel	possibly-damaging	1 (0.7)	69y-77y	T/C	ε3ε4	EXON 13	0	-	-
		21:27326907	c.1684 G>A	p.V562I	A	rs199586073	tolerated	0	-	-	-	EXON 13	1 (0.5)	G/A	ε3ε3
		21:27284167	c.1795 G>A	p.E599K	A	rs140304729	possibly-damaging	0	-	-	-	EXON 14	1 (0.5)	G/A	ε3ε4
	*PSEN2*	1:227071448	c.184 C>T	p.R62C	T	rs150400387	possibly-damaging	1 (0.7)	83y-91y	C/T	ε3ε3	N-Terminal	0	-	-
		1:227071449	c.185 G>A	p.R62H	A	rs58973334	tolerated	1 (0.7)	75y-89y	G/A	ε3ε3	N-Terminal	0	-	-
		1:227073271	c.389 C>T	p.S130L	T	rs63750197	possibly-damaging	1 (0.7)	69y-77y	C/T	ε3ε3		1 (0.5)	C/T	ε2ε2
		1:227083249	c.1316 A>C	p.D439A	C	rs63750110	possibly-damaging	1 (0.7)	75y-89y	A/C	ε3ε3	C-Terminal	1 (0.5)	A/C	ε3ε3
	*GRN*	17:42428954	c.970 G>A	p.A324T	A	rs63750541	tolerated	0	-	-	-		2 (1.1)	G/A	ε3ε3, ε3ε2
		17:42429497	c.1294 C>T	p.R432C	T	rs63750130	tolerated	1 (0.7)	94y	C/T	ε3e4		0	-	-
		17:42429500	c.1297 C>T	p.R433W	T	rs63750412	possibly-damaging	1 (0.7)	69y-81y	C/T	ε4ε4		0	-	-
		17:42430128	c.1744 G>A	p.A582T	A	rs72824737	tolerated	0	-	-	-		1 (0.5)	G/A	ε3ε3
	*MAPT*	17:44068824	c.115-2A>T	frameshift	T	novel	possibly-damaging	1 (0.7)	81y-89y	A/T	ε4ε4		0	-	-
		17:44060841	c.671 T>G	p.V224G	G	rs141120474	possibly-damaging	2 (1.4)	74y-82y; 88y-	T/G	ε2ε3; ε2ε3		1 (0.5)	T/G	ε3ε2
		17:44060807	c.637 G>A	p.G213R	A	rs76375268	possibly-damaging	2 (1.4)	74y-82y; 75y-	G/A	ε3ε4; ε3ε3		0	-	-
		17:44060769	c.599 G>A	p.G200E	A	novel	possibly-damaging	0	-	-	-		1 (0.5)	G/A	ε3ε3
	*PRNP*	20:4680266	c.400 A>G	p.M134V	G	novel	possibly-damaging	0	-	-	-		1 (0.5)	A/G	ε3ε2
*LIKELY LOW FREQUENCY AND COMMON BENIGN POLYMORPHISMS*															
	*PRNP*	20:4680094-4680118		delACAGCCTCATGGTGGTGGCTGGGG	delACAGCCTCATGGTGGTGGCTGGGG	rs138688873	possibly-damaging	2 (1.4)	80y-88y; 76y-83y	delACAGCCTCATGGTGGTGGCTGGGG	ε3ε3; ε3ε3		0	-	-
		20:4680251	c.385 A>G	p.M129V	G	rs1799990	tolerated	64 (45)		A/G			68 (38)	A/G	

Rare variants in causative genes for the monogenic forms of neurodegenerative dementias (AD, FTD, PSP, CBD, CJD): amyloid precursor protein, *APP* (NM_000484.3); presenilins 1and 2, *PSEN1* (NM_000021.3) and *PSEN2* (NM_000447.2); progranulin, *GRN* (NM_002087.2); microtubule associated protein Tau, *MAPT* (NM_001123066.3); prion protein, *PRNP* (NM_000311.3).

Key: AD, Alzheimer’s disease; AAD, age at death; AAO, age at onset; CJD, Creutzfeldt-Jakob disease; FAD, familial Alzheimer’s disease; FTD, frontotemporal dementia; PSP, progressive supranuclear palsy; Aa, amino acid.

* Classification based on the algorithm proposed by Guerreiro et al., 2010a.

*PRNP* and *APP* harbor an higher relative proportion of rare coding variants in controls (1.3/Kb and 1.2/Kb, respectively), compared to cases (0/Kb and 0.4/Kb, respectively), thus, suggesting that rare coding variability in these genes may be well tolerated (Table 3). On the other hand, no controls carry any rare variant in *PSEN1*, suggesting that an amino acid change in PS1 is frequently pathogenic.

**Table 3:** Relative frequency of rare variants (rare variants for Kb of coding sequence) in late-onset AD (LOAD) cases and controls (CTRLS) in *APP*, *PSEN1*, *PSEN2*, *MAPT*, *GRN*, *PRNP*

Gene	LOAD cases (n = 141)	CTRLS (n = 179)
*APP*	0.4/Kb	1.2/Kb
*PSEN1*	0.6/Kb	0/Kb
*PSEN2*	3.7/Kb	1.5/Kb
*MAPT*	1.3/Kb	0.8/Kb
*GRN*	1.1/Kb	1.1/Kb
*PRNP*	0/Kb	1.3/Kb

In our LOAD cohort, we identified a total of 9 rare coding variants in *APP*, *PSEN1*, *PSEN2*, *MAPT*, *GRN*, and *PRNP*, absent in controls. Of these, 2 are likely to be pathogenic, one in *PSEN1* (p.I168T) and the other in *PSEN2* (p.A237V). In contrast, the variants detected in *APP*, *GRN*, *MAPT*, and *PRNP* are likely tolerated polymorphisms.

Several lines of evidence suggest that p.I168T in *PSEN1* is a deleterious change. First, it clusters in the third transmembrane domain (TM3), on the alpha helix surface, where all the known pathogenic variants have been reported (alpha-helix rule) (Hardy and Crook, 2001). Second, a 4 bp inframe deletion (g.38798_38800delTAT, ΔI167; ΔI168) has already been described in a British family with early-onset Alzheimer's disease (Janssen et al., 2003). The patient carrying this variant (*PSEN1* p.I168T) was diagnosed at 86 years of age, heterozygous for *APOE* ε4 allele (ε2ε4), presented an advanced Alzheimer's disease (Braak V), and did not report any positive family history.

The *PSEN2* p.A237V has been only recently reported by the ClinSeq pilot study (Biesecker et al., 2009) and is likely to be a functional variant with a probable deleterious effect. It clusters on the alpha helix surface of the fifth transmembrane domain (TM5), corresponds to a conserved residue among different species and in *PSEN1* (p.A231), where 2 causative mutations (p.A231V and p.A231T) have been described in a Dutch, French and Canadian family (Campion et al., 1999, Cruts et al., 1998, Rogaeva et al., 2001). The patient carrying the p.A237V variant was diagnosed at 87 years, homozygous for *APOE* ε3 allele, and did not refer any family history of AD.

The other variants detected in our cohort in *PSEN2* (p.R62C, p.R62H, p.S130L, and p.D439A) do not cluster in any TM domain. In addition, the p.S130L and p.D439A have been found also controls.

*GRN* harbors 4 missense mutations (p.A324T, p.R432C, p.R433W, and p.A582T), 2 of which have only been detected in cases (p.R432C and p.R433W). Although the p.R432C variant has been already associated with familial FTD and clinical AD (Brouwers et al., 2008, Cruchaga et al., 2012, Shankaran et al., 2008), its pathogenic role remains unclear. By contrast, the p.R433W has been reported as a nonpathogenic variant (www.molgendatabase).

The variants detected in *MAPT* (c.115-2A>T, p.V224G, p.G213R and p.G200E) cluster outside the microtubule binding domain, where most of the pathogenic mutations have been reported up to date (www.molgendatabase). The c.115-2A>T is predicted to alter Tau exon 7 splicing, introducing a nonsense codon within exon 11. We have not detected any difference in exon 7 and in *MAPT* expression between the splice-site mutation carrier and the other cases and controls. Thus, these findings suggest that c.115-2A>T is not disease related (Supplementary data).

The *MAPT* p.G213R has been found in 2 cases and is absent from the controls. It has been described as possibly damaging by *in silico* predictions and clusters close to a pivotal phosphorylation site (S214) for the serum and glucocorticoid inducible kinase 1 (SGK1). SGK1 controls neurite outgrowth by depolarizing the microtubules through the serine phosphorylation at codon 214 (Yang et al., 2006).

Two cases and no controls carry a 24 bp deletion in the *PRNP* open reading frame (rs138688873), between the repeat 3 (R3) and repeat 4 (R4). This octapeptide deletion (rs138688873) has been described as a risk factor for neurodegenerative diseases with some controversy (Palmer et al., 1993, Perry et al., 1995). Furthermore, although these 2 patients were homozygous for *PRNP* p.M129M, a common polymorphism and risk factor for sporadic Creutzfeldt-Jakob disease (sCJD) (Palmer et al., 1991), p.M129M was not significantly associated with AD in this study (*p*-value = 0.22, OR = 0.75). Thus, suggesting this common variant may be a benign polymorphism.

Finally, we report a significant association between *APOE* ε4 allele and LOAD in our cohort (*p*-value = 0.0002, OR = 2.4). Nevertheless, we have not detected any enrichment for the *APOE* ε4 allele among the patients carrying rare variants in *APP*, *PSEN1*, *PSEN2*, *GRN*, *MAPT*, and *PRNP* (only 6 of 14 LOAD carriers [42%] carry at least one *APOE* ε4 allele). By contrast, *APOE* ε2 allele did not present a significantly higher frequency in controls compared to cases (*p*-value = 0.3, OR = 0.66).

## 4 Discussion

In this study, we tested the hypothesis that the significant phenotypic overlap between sporadic LOAD and other neurodegenerative dementias (FAD, FTD, PSP, CBD, and CJD) may be explained by a common genetic background. Thus, we screened 6 Mendelian dementia genes (*APP*, *PSEN1*, *PSEN2*, *MAPT*, *GRN*, and *PRNP*) aiming to establish whether rare coding variability in these genes is responsible for an appreciable portion of the LOAD risk.

In our LOAD cohort, we found a novel rare variant in *PSEN1* (p.I168T) and a rare variant in *PSEN2* (p.A237V). These variants are likely pathogenic: (1) both cluster in TM domains, on the alpha helix surface; (2) the literature already reported in the same (*PSEN1* p.I168) or homologous residue (*PSEN2* p.A237 and *PSEN1* p.A231) causative mutations for FAD; and (3) the *PSEN1* p.I168T and *PSEN2* p.A237V are classified as possible pathogenic, following the algorithm proposed by Guerreiro et al. (2010a). The other variants detected in our LOAD cases are likely to be tolerated. First, they have already been described as benign polymorphisms (*PSEN2* p.R62H; *PRNP* rs138688873 and *GRN* p.R433W) (Guerreiro et al., 2010a, Palmer et al., 1993; www.molgendatabase). Second, they cluster outside the reported pathogenic domains (*APP* p.A201V, p.Y538H, p.V562I, p.E599K; *PSEN2* p.R62C, p.R62H, p.S130L, p.D439A; *MAPT* p.V224G, p.G213R and p.G200E). Third, they do not alter the gene expression (*MAPT* c.115-2A>T).

Finally, despite the functional consequence of *GRN* p.R432C, the effect of a decreased *GRN* secretion in AD pathogenesis remains controversial. *GRN* pathogenic mutations act through a messenger RNA nonsense-mediate decay, interfering with *GRN* expression and generally are loss of function mutations (LoF) (stop-gain, frameshift mutations, and deletions). The only exception to this rule is represented by the pathogenic missense mutations which cluster in the GRN signal peptide domain (*GRN* p.A9D) (www.molgendatabase). Thus, we suggest, in concert with previous studies (Guerreiro et al., 2010b), that *GRN* missense mutations mapping outside the signal peptide domain are likely to be well tolerated.

In conclusion, our findings support recent studies, suggesting that rare coding variability in *PSEN1* and *PSEN2* contributes to susceptibility for apparently sporadic LOAD. Therefore, sporadic LOAD and FAD may be influenced by the same genes and thus pathogenic mechanisms. On the contrary, rare coding variants in *MAPT*, *GRN*, and *PRNP* are not major players in the development of LOAD. Thus, genetic screening is fundamental for the differential diagnosis of these disparate neurodegenerative dementias.
