# Supplementary material for: Investigating the role of rare coding variability in Mendelian dementia genes (APP, PSEN1, PSEN2, GRN, MAPT, and PRNP) in late-onset Alzheimer's disease
Source: Neurobiol Aging. 2014 Dec;35(12):2881.e1–6. doi: 10.1016/j.neurobiolaging.2014.06.002 (PMC4236585; doi:10.1016/j.neurobiolaging.2014.06.002)
Supplement: Supplementary Material [file mmc1.docx]

*Investigating the role of rare coding variability in Mendelian dementia genes (APP, PSEN1, PSEN2, GRN, MAPT and PRNP) in late onset Alzheimer’s disease*

Sassi C.^1,2^, Guerreiro R.^1,2^, Gibbs R.^1,2^, Ding J.^2^, Lupton M.K^3^, Troakes C ^3^, Al-Sarraj S. ^3^, Niblock M^3^., Gallo J-M^3^, Adnan, J.^3^, Killick R.^3^, Brown K. S.^4^, Medway C.^4^, Lord J.^4^, Turton J.^4^, Bras J.^1^, ARUK Consortium*, Morgan K.^4^, Powell J.F.^3^, Singleton A.^2^, Hardy J.^1^.

*The Alzheimer’s Research UK (ARUK) Consortium: Peter Passmore, David Craig, Janet Johnston, Bernadette McGuinness, Stephen Todd, Queen’s University Belfast, UK; Reinhard Heun, Royal Derby Hospital, UK; Heike Kölsch, University of Bonn, Germany; Patrick G. Kehoe, University of Bristol, UK; Nigel M. Hooper, University of Leeds, UK; Emma R.L.C. Vardy, University of Newcastle, UK; David M. Mann, Stuart Pickering-Brown, University of Manchester, UK; Kristelle Brown, James Lowe, Kevin Morgan, University of Nottingham, UK; A. David Smith, Gordon Wilcock, Donald Warden, University of Oxford (OPTIMA), UK; Clive Holmes, University of Southampton, UK.

^1^ Department of molecular Neuroscience, UCL Institute of Neurology, University College London, London, UK

^2^ Laboratory of Neurogenetics, National Institute on Aging, National Institutes of Health, Bethesda, MD, USA

^3^ King’s College London Institute of Psychiatry, London, United Kingdom

^4^ Translation Cell Sciences-Human Genetics, School of Life Sciences, Queens Medical Centre, University of Nottingham, Nottingham, United Kingdom

**Supplementary material**

**Additional functional data for the c.115-2A>T Tau variant.**

1. **Introduction**

The c.115-2A>T Tau variant is predicted to affect splicing of exon 7 which is a constitutive exon and loss of this exon would lead to a frameshift and result in a premature termination codon (PTC) within exon 11. As brain tissue was available for the patient carrying the *MAPT* c.115-2A>T variant , it was possible to determine (a) if the mutation causes loss of *MAPT* exon 7 and (b) if the potential loss of the exon causes a PTC and results in a reduction in *MAPT* transcripts by nonsense mediated decay.

1. **Material and methods**

*2.1 MAPT expression study*

We assessed expression of Tau exon 7 by end-point RT-PCR. RNA was extracted from the temporal cortex and RNA integrity was measured with the Agilent RNA 6000 pico kit using the Agilent 2100 Bioanalyser according to the manufacturer’s protocol. A RIN of 3.6 and above was found to be sufficient integrity for RT-PCR analyses of human brain RNA. 1 µg of RNA was reverse transcribed with oligo(dT) and reagents from the Taqman RT kit (G E Healthcare). PCR was performed with GoTaq polymerase and reagents from Promega.

A forward primer (5’ to 3’) GCATGGTCAGTAAAAGCAAAGACGG 3’ which binds to a sequence in Tau exon 5 and a reverse primer GCTCTTGGCGGAAGACGGC (5’ to 3’) which binds to a sequence in exon 9 were used.

Cycling parameters

Cycles 30

Initial separation 5 mins 95°C

Denaturation 30 secs 95°C

Annealing 20 secs 57°C

Extension 30 secs 72°C

Final extension 10 mins 72°C

Additionally, total levels of Tau transcripts were measured by qRT-PCR. RNA from the temporal cortex was extracted and the RIN was determined as above for AD (n = 24) and control (n= 13) samples. RNA was reverse transcribed as above and 20 ng of cDNA was used for each reaction. qRT-PCR was carried out in 384 well plates using the ABI 7900HT (Applied Biosystems) system. The two most stable reference genes (*GAPDH* and *CYC1*) were identified with primers and software (qbase^PLUS^) from Primerdesign (UK). Ct values were measured in duplicate for Tau, GAPDH and CYC1 and an average CT for each was calculated. The average Tau Ct was divided by the geometric mean of the two reference genes and each point plotted giving *MAPT* gene expression relative to reference gene expression (**Figure S1 B**).

The primers for qRT PCR primers were: forward (5’ to 3’) CCATCATAAACCAGGAGGTGGCC which spans Tau exons 11 and 12; reverse (5’ to 3’) GGTCAGCTTGTGGGTTTCAATCTT which spans exons 12 and 13. The primer pair produces a 148 bp PCR product which was sequenced and shown to produce a single product in qRT-PCR reactions.

Cycling parameters for qRT-PCR

Initial separation 10 mins 95°C

Denaturation 15 secs 95°C

Annealing 30 secs 57°C

Extension 15 secs 72°C

*2.2 Immunoblotting*

Human brain tissue (superior temporal gyrus) was obtained from the MRC London Neurodegenerative Disease Brain Bank, King’s College London. Frozen tissues were thawed on ice in an 8 x wt/vol amount of PBS-based homogenisation buffer containing phosSTOP and miniComplete, phosphatase and protease inhibitor cocktails (Roche, Sussex, UK), 25 mM β-glycerophosphate and 25 mM NaF, homogenised using a glass homogeniser and centrifuged at 100,000g (Beckman Coulter (UK) Ltd, High Wycombe, UK). Supernatants were collected and proteins separated by SDS-PAGE in 10% gels and immunoblotted for Tau using antibody A0024 (Dako UK Ltd, Ely, UK) and V-20 (sc-1996 - Santa Cruz Biotechnology Inc., Santa Cruz, USA). Immunoblots were imaged using a Li-Cor Odyssey infrared scanner (LiCor Biosciences, Lincoln, USA) as previously described (Killick et al., 2009).

1. **Results**

Amplification with primers annealing with Tau exon 5 and exon 9 generated a 347 base pair product from cDNA generated from human brain samples RNA. The identity of the product was confirmed by sequencing. There was no detectable differences in levels of this PCR product for the sample carrying the *MAPT* c.115-2A>T variant compared to other AD samples or controls (**Figure S1 A**) and no additional product corresponding to an exon 7 deletion was observed. Quantitative RT-PCR analysis showed that the *MAPT* intron 7 variant had an expression level within the normal range for both AD and controls (see arrow in **Figure S1 B** showing the Tau intron 7 variant data point). There were no significant differences in MAPT expression between the AD group compared to controls.

To determine if the *MAPT* variant affects the pattern of Tau proteins, human brain samples were analysed to immunoblotting for Tau. Lysates from three controls, three AD cases and the LOAD case harboring the *MAPT* exon 7 variant (c.115-2A>T) were probed with an antibody, which recognises a non-phosphorylation sensitive epitope in the C-terminal portion of the protein and detects all 6 major isoforms generated by alternate splicing (Dako - A0024). A second antibody recognises an epitope N-terminal of the variant, was also used. No changes in Tau pattern was observed with either antibody (**Figure S1 C**, left and right panels).

1. **Discussion**

No evidence was found to support the *in silico* prediction that the c.115-2A>T variant affected splicing of the Tau pre-mRNA.

**FIGURES**


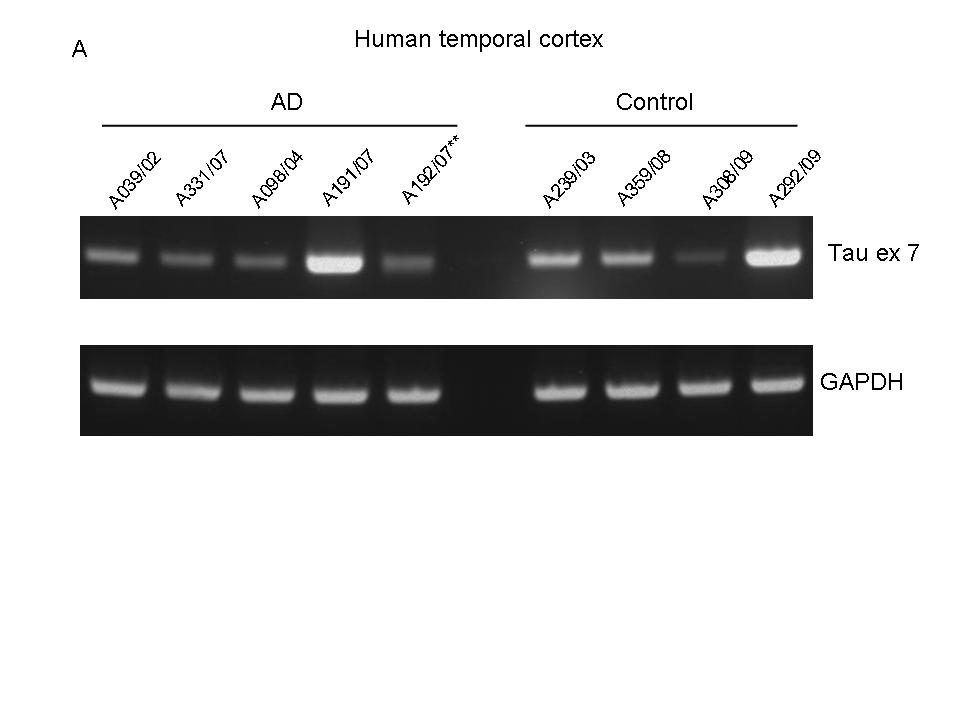


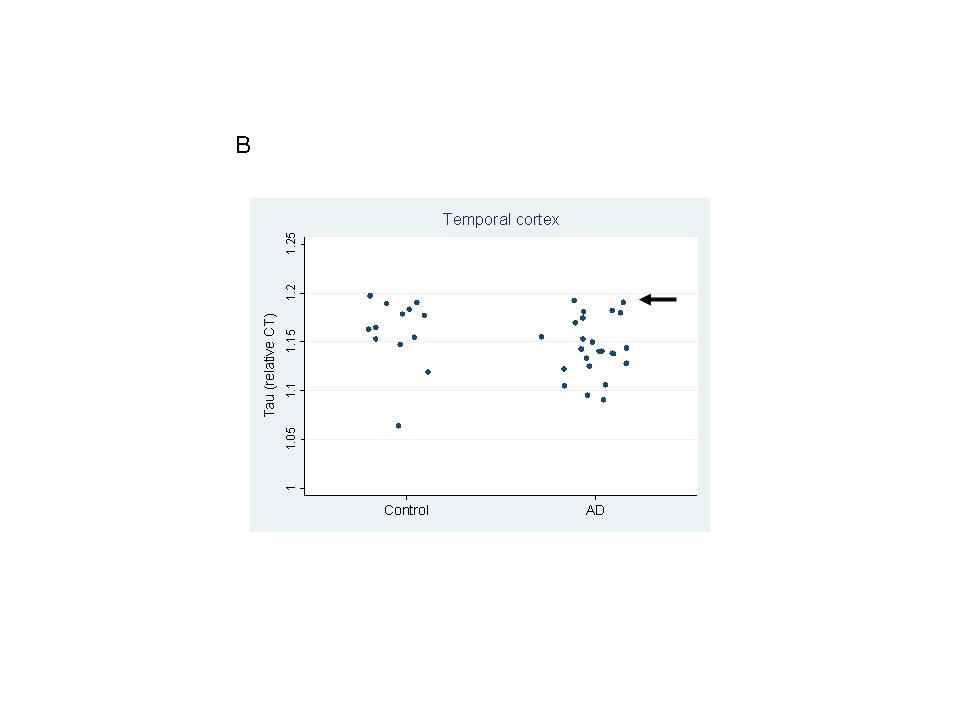


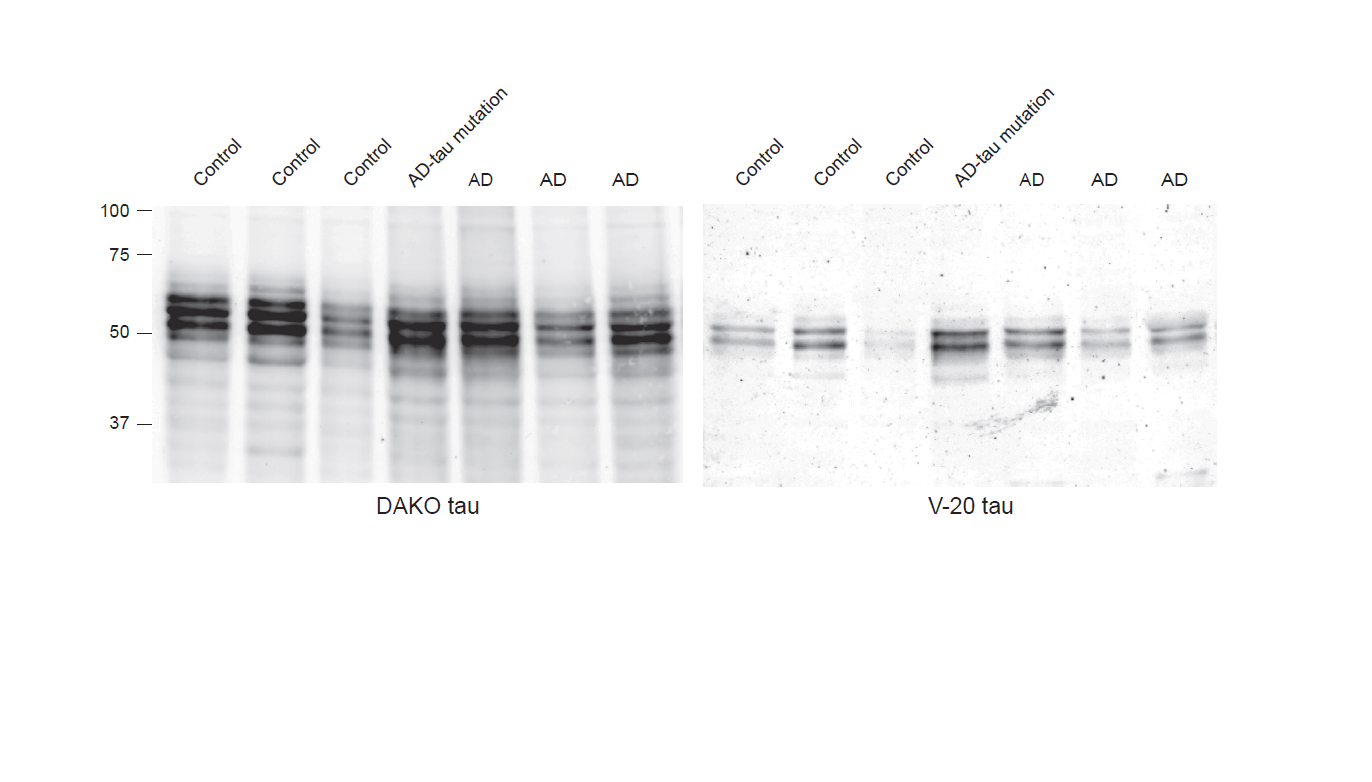


C

**Figure S1 *MAPT* c.115-2A>T, expression study**. **A)** **RT-PCR** . No difference in Tau PCR product including exon 7 between the splice-site variant carrier (A192/07**) and other AD cases and controls were detected. GAPDH has been used as a control. **B)** Critical threshold (Ct) values were measured in duplicate for Tau and two reference genes (*GAPDH* and *CYC1*) and an average Ct for each was calculated. No significant differences in *MAPT* expression was detected in the AD group compared to controls. **C)** Immunoblotting for Tau

**References**

Killick, R., [Scales G](http://www.ncbi.nlm.nih.gov/pubmed?term=Scales%20G%5BAuthor%5D&cauthor=true&cauthor_uid=19523444), [Leroy K](http://www.ncbi.nlm.nih.gov/pubmed?term=Leroy%20K%5BAuthor%5D&cauthor=true&cauthor_uid=19523444), [Causevic M](http://www.ncbi.nlm.nih.gov/pubmed?term=Causevic%20M%5BAuthor%5D&cauthor=true&cauthor_uid=19523444), [Hooper C](http://www.ncbi.nlm.nih.gov/pubmed?term=Hooper%20C%5BAuthor%5D&cauthor=true&cauthor_uid=19523444), [Irvine EE](http://www.ncbi.nlm.nih.gov/pubmed?term=Irvine%20EE%5BAuthor%5D&cauthor=true&cauthor_uid=19523444), [Choudhury AI](http://www.ncbi.nlm.nih.gov/pubmed?term=Choudhury%20AI%5BAuthor%5D&cauthor=true&cauthor_uid=19523444), [Drinkwater L](http://www.ncbi.nlm.nih.gov/pubmed?term=Drinkwater%20L%5BAuthor%5D&cauthor=true&cauthor_uid=19523444), [Kerr F](http://www.ncbi.nlm.nih.gov/pubmed?term=Kerr%20F%5BAuthor%5D&cauthor=true&cauthor_uid=19523444), [Al-Qassab H](http://www.ncbi.nlm.nih.gov/pubmed?term=Al-Qassab%20H%5BAuthor%5D&cauthor=true&cauthor_uid=19523444), [Stephenson J](http://www.ncbi.nlm.nih.gov/pubmed?term=Stephenson%20J%5BAuthor%5D&cauthor=true&cauthor_uid=19523444), [Yilmaz Z](http://www.ncbi.nlm.nih.gov/pubmed?term=Yilmaz%20Z%5BAuthor%5D&cauthor=true&cauthor_uid=19523444), [Giese KP](http://www.ncbi.nlm.nih.gov/pubmed?term=Giese%20KP%5BAuthor%5D&cauthor=true&cauthor_uid=19523444), [Brion JP](http://www.ncbi.nlm.nih.gov/pubmed?term=Brion%20JP%5BAuthor%5D&cauthor=true&cauthor_uid=19523444),[Withers DJ](http://www.ncbi.nlm.nih.gov/pubmed?term=Withers%20DJ%5BAuthor%5D&cauthor=true&cauthor_uid=19523444), [Lovestone S](http://www.ncbi.nlm.nih.gov/pubmed?term=Lovestone%20S%5BAuthor%5D&cauthor=true&cauthor_uid=19523444). , 2009. Deletion of Irs2 reduces amyloid deposition and rescues behavioural deficits in APP transgenic mice. Biochem Biophys Res Commun *386*, 257-262.
